# Revertant Mosaicism in Genodermatoses: Natural Gene Therapy Right before Your Eyes

**DOI:** 10.3390/biomedicines10092118

**Published:** 2022-08-29

**Authors:** Peter C. van den Akker, Maria C. Bolling, Anna M. G. Pasmooij

**Affiliations:** 1 Groningen Center for Blistering Diseases, Department of Genetics, University of Groningen, University Medical Center Groningen, 9700 RB Groningen, The Netherlands; 2 Division of Biological Chemistry and Drug Development, School of Life Sciences, University of Dundee, Dundee DD1 5EH, Scotland, UK; 3 Groningen Center for Blistering Diseases, Department of Dermatology, University of Groningen, University Medical Center Groningen, 9700 RB Groningen, The Netherlands

**Keywords:** genodermatosis, revertant mosaicism, natural gene therapy, epidermolysis bullosa, ichthyosis, gene therapy

## Abstract

Revertant mosaicism (RM) is the intriguing phenomenon in which nature itself has successfully done what medical science is so eagerly trying to achieve: correcting the effect of disease-causing germline variants and thereby reversing the disease phenotype back to normal. RM was molecularly confirmed for the first time in a genodermatosis in 1997, the genetic skin condition junctional epidermolysis bullosa (EB). At that time, RM was considered an extraordinary phenomenon. However, several important discoveries have changed this conception in the past few decades. First, RM has now been identified in all major subtypes of EB. Second, RM has also been identified in many other genodermatoses. Third, a theoretical mathematical exercise concluded that reverse mutations should be expected in all patients with a recessive subtype of EB or any other genodermatosis. This has shifted the paradigm from RM being an extraordinary phenomenon to it being something that every physician working in the field of genodermatoses should be looking for in every patient. It has also raised hope for new treatment options in patients with genodermatoses. In this review, we summarize the current knowledge on RM and discuss the perspectives of RM for the future treatment of patients with genodermatoses.

## 1. Genodermatoses

Genodermatoses are a group of genetic skin conditions caused by pathogenic genomic variants that affect genes expressed in one of the compartments of the skin. Their (birth) prevalence varies widely, with most being rare to extremely rare. Depending on the definition used, more than 500 different types of genodermatoses can be distinguished, for which several hundred causal genes are known (see the Online Mendelian Inheritance in Man database, www.omim.org, accessed 1 June 2022). The phenotypic spectrum of genodermatoses, as well as their severity, covers a broad continuum. On the most severe end are the lethal neonatal or early childhood phenotypes like acantholytic epidermolysis bullosa (EB) (in the group of erosive skin fragility disorders [1]), which is caused by biallelic carboxyl-terminally truncating mutations in the desmosomal protein desmoplakin encoded by the *DSP* gene [2,3], autosomal recessive congenital ichthyosis (ARCI)—harlequin type, which is caused by biallelic null-variants in the *ABCA12* gene coding for the ATP-binding cassette (ABC) A12-transporter protein [4,5], and restrictive dermopathy caused by biallelic null-variants in the *ZMPSTE24* gene, which encodes the zinc metalloproteinase STE24 that is crucial for the processing and maturation of the lamin A protein [6,7]. At the other end of the genodermatosis spectrum are relatively mild and/or late-onset conditions like mild forms of ectodermal dysplasia (ED), which is caused by either homozygous or heterozygous missense variants in the wingless family member 10A gene *WNT10A* [8], and the nails-only type of localized dystrophic EB (DEB) that is caused by glycine substitutions in the type VII collagen encoding gene *COL7A1* [9]. However, the precise phenotype of the multiple genodermatoses varies greatly and depends on the gene involved and the nature of the causative pathogenic variants. Consequently, there is large clinical heterogeneity and variable expression among the genodermatoses, and most disease groups within the spectrum of genodermatoses also have broad clinical spectra. For instance, while biallelic null-variants in *ABCA12* cause the very severe harlequin type of ARCI, ‘milder’ variants, usually missense or splice-site variants, cause a type of ARCI with or without a collodion membrane and erythroderma that is very similar to other forms of ARCI that do not affect life expectancy in most cases [10]. In addition, while heterozygous or even homozygous missense variants in *WNT10A* cause mild forms of ED or isolated hypo- or oligodontia, biallelic truncating variants can cause more severe types of ED like hypohidrotic ED (HED), odonto-onycho-dermal dysplasia, or Schöpf–Schulz–Passarge syndrome [8]. Finally, whereas certain heterozygous glycine substitutions in *COL7A1* only affect the halluces [9], biallelic null-variants in *COL7A1* are associated with a very severe type of recessive DEB (RDEB) with severe morbidity and early mortality [11]. In addition to clinical heterogeneity, there is also genetic heterogeneity among the genodermatoses. For instance, variants in 13 genes are now known to be implicated in one type of ARCI (www.omim.org, accessed on 1 June 2022), with largely overlapping features [12].

## 2. Mosaicism in Genodermatoses

**Figure 1:**
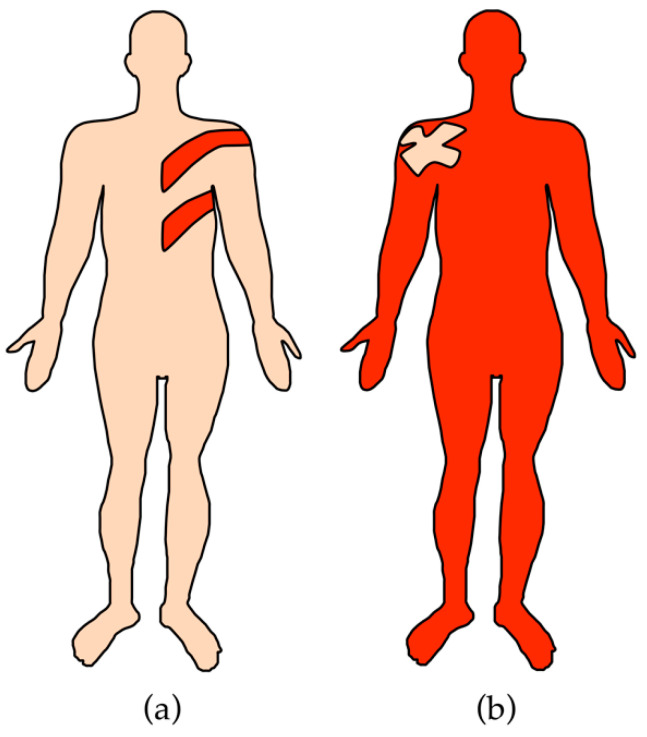
Comparison of forward and revertant mosaicism in genodermatoses. (**a**) In forward mosaicism, a somatic variant occurring on a wildtype allele induces a mosaic disease phenotype amidst otherwise healthy skin. (**b**) In revertant mosaicism, a somatic variant corrects the effect of a germline variant leading to the correction of the disease phenotype in a mosaic distribution. Light pink: healthy skin. Red: affected skin.

Another important disease-modifying phenomenon is somatic mosaicism of the causal gene variants. Somatic mosaicism is the coexistence, in one individual, of cell lines that are (epi)genetically different yet originated from a single zygote [13]. The proportions of the cell lines within the body determine the extent and severity of the associated disease phenotype and, in genodermatoses, often result in skin areas with different disease expression [14]. Mosaicism is usually due to a somatic genetic or epigenetic change that occurred post-zygotically in one of the cells of the otherwise genetically homogeneous developing embryo [13]. A recent review proposed classifying mosaicism according to six different attributes (A–F): **A**ffected tissue (somatic and/or germinal), **B**ody pattern (segmental vs. non-segmental), **C**hange of direction (‘forward’ vs. ‘revertant’), **D**evelopmental mechanism (type I vs. type II segmental mosaicism, functional X-linked mosaicism, disorders only manifesting as mosaics), **E**tiology (type of genomic alteration), and the **F**raction of affected tissue (mild–severe involvement) [15]. In this review, we will only discuss mosaicism based on the direction of a somatic genetic alteration, i.e., ‘revertant mosaicism’ (RM), and only briefly discuss ‘forward mosaicism’ as a comparison (Figure 1).

## 3. Forward Mosaicism in Genodermatoses

In forward mosaicism, a spontaneous somatic variant mutates a wildtype allele, thereby inducing a disease phenotype in the cell population where it occurs. For a recent review on forward mosaicism and the challenges in properly diagnosing it at the molecular level, we refer the reader to Cheraghlou et al. [14]. In cases where the somatic mutation occurs in a cell containing two wildtype alleles and the gene involved is associated with a dominantly inherited disease, the heterozygous cell populations will express a mosaic phenotype of the dominant genodermatosis. Many examples of this ‘type 1 segmental’ mosaicism have been described, e.g., in segmental neurofibromatosis type 1 or mosaic RASopathy-associated epidermal nevi [16,17]. This group also includes the disorders that can only manifest in a mosaic state because the autosomal dominant, and usually activating, variants are lethal if present in an entire body, e.g., McCune–Albright syndrome due to the somatic *GNAS* variants p.(Arg201His) or p.(Arg201Cys) [18], and *PIK3CA*-related overgrowth spectrum [19,20].

A similar event can occur in cells that already carry a mutant allele of a gene involved in an autosomal recessive genodermatosis. The affected cell populations will then carry biallelic gene variants and express a disease phenotype in a mosaic fashion. Remarkably, while there is a large number of autosomal recessive genodermatoses and there are millions of carriers of the gene variants in the associated genes, segmental type 1 mosaicism for such recessive genodermatoses is only rarely reported. One of the few examples is a case of blaschkoid congenital ichthyosiform erythroderma due to somatic mosaicism for a second *ABCA12* variant [21]. While this has not been thoroughly investigated, this lack of observations may reflect that, for many disorders, cells lacking the protein encoded by the gene involved have a developmental disadvantage.

Forward mosaicism can also occur on the wildtype allele in cells that already carry a heterozygous mutation for an autosomal dominant or X-linked (in women) genodermatosis. This loss of heterozygosity for the wildtype allele either increases the severity of the disease in the affected cell populations or changes the nature of the disease, leading to type 2 segmental mosaicism [22]. Type 2 segmental mosaicism has been reported in many genodermatoses, e.g., Darier disease, which is caused by variants in the *ATP2A2* gene, or Gorlin syndrome, caused by variants in the *PTCH1* gene [23,24]. Irrespective of the type of mosaicism or the disorder involved, the expression of the somatic phenotype is always determined by the cell type and proportion of cells that carry the somatic variant, the timing of its occurrence, and the nature and effect of the somatic variants [13,14,15].

## 4. Revertant Mosaicism in Genodermatoses

In RM, an additional somatic change mitigates the effect of the germline variant(s) and the direction of mosaicism is towards improvement rather than the induction or worsening of the disease phenotype [15]. In 1995, Jonkman et al. described a patient with the type XVII collagen-deficient intermediate type of junctional EB (JEB) due to germline *COL17A1* mutations who exhibited normal-looking, non-affected skin areas in which blistering could not be induced by rubbing [25]. Skin biopsy sections showed focal areas of positive type XVII collagen-staining, indicating RM in a micro-mosaic pattern. In 1997, the presence of RM in this patient was proven at the DNA level, making it the first confirmed case of RM in a genodermatosis [26], although RM had already been described in other genetic conditions (Supplementary Table S1) [27,28,29,30,31,32,33,34,35,36,37,38,39,40,41,42,43,44,45,46,47,48,49,50,51,52,53,54,55,56,57,58,59,60,61]. In fact, RM had been demonstrated as early as 1977 in patients with Bloom’s syndrome through the observation of coexistence of cells with a greatly increased number of sister chromatid exchanges next to cells with a normal number [62]. However, RM could not be proven at the DNA level at that time due to the lack of appropriate techniques. The first case of RM proven at the DNA level was a patient with the X-linked recessive condition, Lesch–Nyhan syndrome [61]. Despite carrying a germline deleterious intragenic duplication of exons 2 and 3 of the *HPRT* gene, this patient had an unusually mild presentation as he did not have the severe intellectual disability that is a common feature of the syndrome. This unusually mild presentation was likely explained by the presence of revertant clones that had lost the duplicated genomic region due to a postzygotic gene rearrangement.

**Figure 2:**
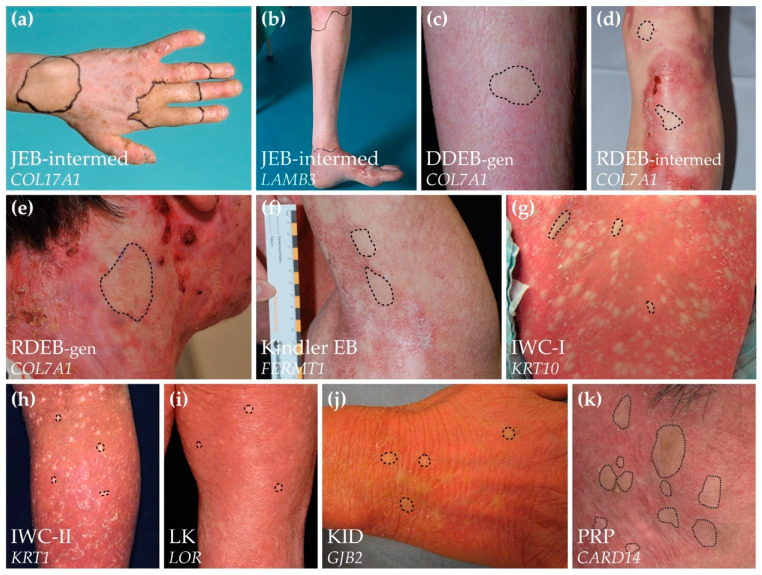
Overview of the revertant skin phenotypes in different genodermatoses. Revertant skin patches in (**a**) the *COL17A1*-associated intermediate type of junctional epidermolysis bullosa (JEB-intermed) [75], (**b**) the *LAMB3*-associated intermediate type of junctional epidermolysis bullosa (JEB-intermed) [74], (**c**) the generalized type of dominant dystrophic epidermolysis bullosa (DDEB-gen, *COL7A1*) [86], (**d**) the intermediate type of generalized recessive dystrophic epidermolysis bullosa (RDEB-intermed, *COL7A1*) [86], (**e**) the severe type of generalized recessive dystrophic epidermolysis bullosa (RDEB-gen, *COL7A1*) [68], (**f**) Kindler epidermolysis bullosa (Kindler EB, *FERMT1*) [70], (**g**) ichthyosis with confetti type 1 (IWC-I, *KRT10*) [81], (**h**) ichthyosis with confetti type 2 (IWC-II, *KRT1*) [87], (**i**) loricrin keratoderma (LK, *LOR*) [83], (**j**) keratitis-ichthyosis-deafness syndrome (KID, *GJB2*), and (**k**) pityriasis rubra pilaris (PRP, *CARD14*) [84]. Solid or dashed lines indicate revertant patches amidst affected skin (note that on several panels more revertant patches are visible than highlighted). Note that the revertant patches in the different types of recessive EB are generally larger than in the autosomal dominant ichthyotic disorders, whereas multiple revertant spots are usually identified simultaneously in the latter. All images re-used with permission and the original articles are cited.

At the time of the initial description of RM in a genodermatosis in 1997, RM was considered an extraordinary phenomenon. However, since that initial description, RM has been identified in all major types of EB (Table 1) [63,64,65,66,67,68,69,70,71,72,73,74,75,76,77,78], and, in 2012, we were able to confirm revertant skin patches in all ten Dutch patients with the intermediate type of JEB due to pathogenic *COL17A1* variants [79]. Around the same time, Choate et al. demonstrated that each of the multiple healthy ‘confetti-like’ spots in patients with ichthyosis with confetti (IWC) due to germline variants in *KRT10* (IWC-I) or *KRT1* (IWC-II) represent a separate occurrence of RM in a single keratinocyte clone [80,81]. Moreover, RM was identified in a patient with keratitis-ichthyosis-deafness (KID) syndrome due to a heterozygous dominant-negative variant in the *GJB2* gene [82]. Suzuki et al. reported that RM also frequently occurs in loricrin keratoderma (LK) [83], and Miyauchi et al. recently reported multiple revertant skin spots in two patients with pityriasis rubra pilaris (PRP) due to gain-of-function missense variants in *CARD14* [84]. Figure 2 provides an overview of the revertant skin phenotype in these genodermatoses. Finally, by applying a mathematical developmental model to the occurrence of RM in patients with recessive types of EB, we concluded that reverse mutations should occur multiple times in the skin of all patients with recessive types of EB [85]. This conclusion corroborates the clinical findings that, contrary to previous conceptions, RM is to be expected in all patients with EB and other genodermatoses. The reason why RM is frequently seen in skin likely has to do with the facts that skin can easily be explored visually and is a rapidly self-renewing organ in which a large number of cell divisions take place, which means it is analogous to bone marrow and the frequent occurrence of RM observed in hematologic and immunologic disorders (Supplementary Table S1).

Our mathematical developmental model also revealed that, to be able to grow to the size of a clinically recognizable patch, RM should occur very early in embryogenesis [85]. However, the chance that a reverse mutation occurs at such an early embryonic stage was calculated to be so low that a revertant patch would be expected in only 1 in 10,000 patients, which clearly underestimates the clinical situation. The only likely explanation was that reverse mutations do occur later during (embryonic) life but impose a significant selective growth advantage on revertant cells during a particular time-window, the “late-but-fitter revertant cell” hypothesis. In KID syndrome, the revertant variants identified *in cis* with the germline variant were also thought to provide the host cells with a selective growth advantage [82]. The UV-signature of the revertant patches in KID syndrome underscores their occurrence later in life and the need for a selective growth advantage. This finding, together with the frequent occurrence of revertant confetti-like spots in IWC and LK during childhood or adolescence that have a specific period of growth followed by stabilization at a maximal size [80,81,83,88,89,90,91] seems to support the “late-but-fitter revertant cell” hypothesis.

**Table 1:** Genetic correction mechanisms of revertant mosaicism reported in genodermatoses.

Disorder		Corrected Gene	Correction Mechanisms				Corrected Germline Mutations	Skin Layer	References
Subtype (MIM)	Inheritance	Name (MIM)	Major Group	Specific Type	Consequence RNA-Level	Consequence Protein-Level			
Epidermolysis bullosa									
EBS, severe (131760)	AD	*KRT14* (148066)	Second-site mutation	Nucleotide insertion (1 Nt)	Disruption of reading frame Silencing dominant negative allele	Loss of expression of mutant protein	c.373C>T; p.Arg125Cys	EKCs	[76]
EBS, recessive (601001)	AR	*KRT14* (148066)	Unknown	Unknown	Splice-modulating Generation of in-frame splice variant	Introduction of protein lacking 2 AA and carrying 1 missense AA. Non-functional.	c.526-2A>C (SS)	EKCs	[77]
JEB, intermediate (226650)	AR	*COL17A1* (113811)	Gene conversion	N/A	Loss of heterozygosity for one mutant allele	Introduction of full-length protein, wildtype	c.1601delA; p.Asp534fs	EKCs	[26,75]
			Back-mutation/ mitotic recombination	Nucleotide change	Nonsense to wildtype reversion	Introduction of full-length protein, wildtype	c.3676C>T; p.Arg1226*	EKCs	[75]
			Second-site mutation	Nucleotide change	Nonsense to missense change	Introduction of full-length protein carrying 1 missense AA	c.3676C>T; p.Arg1226*	EKCs	[75]
					Splice-modulating Restoration of reading frame	Introduction of full-length protein with 13 incorrect AA	c.4319dup; p.Gly1441fs	EKCs	[75]
					In-frame skipping of mutant exon	Introduction of shorter protein lacking AA of mutant exon	c.2237del; p.Gly746fs	EKCs	[73,79]
							c.3487G>T; p.Glu1163*	EKCs	[66]
				Nucleotide insertion (2 Nt)	Restoration of reading frame	Introduction of full-length protein with 25 incorrect AA	c.3899_3900del; p.Ser1300fs	EKCs	[78]
				Intragenic genomic deletion	In-frame skipping of mutant exon	Introduction of shorter protein lacking AA of mutant exon	c.2237del; p.Gly746fs	EKCs	[73,79]
					In-frame deletion of multiple exons	Introduction of shorter protein lacking AA of mutant exon	c.2237del; p.Gly746fs	EKCs	[73,79]
				Intragenic genomic deletion and insertion	In-frame deletion of mutant exon	Introduction of shorter protein lacking AA of mutant exon	c.2237del; p.Gly746fs	EKCs	[73,79]
		*LAMB3* (150310)	Second-site mutation	Nucleotide change	Splice-modulating. Increase in wild-type splicing pattern	Increased expression of full-length protein carrying 1 missense mutation	c.628G>A; p.Glu210Lys	EKCs	[74]
					Splice modulating. Introduction of new, in-frame splice variant	Introduction of protein elongated by 22 AA	c.628G>A; p.Glu210Lys	EKCs	[74]
					Splice modulating. Increased expression of alternative in-frame splice variant	Increased expression of protein lacking 22 AA	c.628G>A; p.Glu210Lys	EKCs	[74]
RDEB, severe (226600)	AR	*COL7A1* (120120)	Intragenic cross-over (mitotic recombination)	N/A	Loss of heterozygosity of one mutant allele	Introduction of full-length protein, wildtype	c.7786del; p.Gly2596fs	EKCs	[72]
			Second-site mutation	Nucleotide deletion (1 bp)	Restoration of reading frame	Introduction of full-length protein, wildtype	c.6527dup; p.Gly2177fs	EKCs	[71]
				Nucleotide change	Splice-modulating. Restoration of splicing pattern towards wild-type	Increased expression of full-length protein, wildtype	c.2142A>G (SS)	EKCs	[86]
							c.425A>G (SS)	EKCs	[86]
			Back mutation/mitotic recombination	N/A	Loss of heterozygosity of one mutant allele	Introduction of full-length protein, wildtype	c.884del; p.Gly296fs	EKCs	[86]
						Introduction of full-length protein, wildtype, loss of expression of mutant protein	c.6176A>G; p.Glu2059Gly	EKCs	[86]
			Mitotic recombination	N/A	Both variants on one allele	Introduction of full-length protein, wildtype	425A>G (SS)	EKCs	[86]
							c.1837C>T; p.Arg613*	EKCs	[86]
RDEB, intermediate (226600)	AR	*COL7A1* (120120)	Mitotic recombination	N/A	Loss of heterozygosity of one mutant allele	Introduction of full-length protein, wildtype	c.425A>G	EKCs	[86]
			Second-site mutation	Nucleotide change	Nonsense to missense change	Introduction of full-length protein with 1 missense AA	c.6508C>T; p.Gln2170*	EKCs	[68]
			Intragenic cross-over (mitotic recombination)	N/A	Loss of heterozygosity of one mutant allele	Introduction of full-length protein, wildtype, loss of expression of mutant protein	c.6091G>A; p.Gly2031Ser	DFBs	[65]
					Both variants on one allele	Introduction of full-length protein, wildtype, loss of expression of mutant protein	c.5932C>T; p.Arg1978*/ c.8029G>A; p.Gly2677Ser	EKCs	[64]
DDEB (131750)	AD	*COL7A1* (120120)	Back mutation/mitotic recombination	Nucleotide change	Loss of heterozygosity of mutant allele	Loss of expression of mutant protein	c.6127C>A; p.Gly2043Arg	EKCs	[86]
Kindler EB (173650)	AR	*FERMT1* (607900)	Slipped mispairing	Nucleotide deletion (1 bp)	Loss of one mutant allele	Introduction of full-length protein, wildtype	c.676dup; p.Gln226fs	EKCs	[69,70]
							c.456dup; p.Asp153fs	EKCs	[69,70]
Disorders of cornification									
Ichthyosis with confetti type 1, KRT10 (609165)	AD	*KRT10* (148080)	Mitotic recombination	N/A	Loss of heterozygosity of mutant allele	Loss of expression of mutant protein	c.1369G>T (SS)	EKCs	[81]
							c.1373del; p.Ser458fs	EKCs	[90]
							c.1373+1G>A (SS)	EKCs	[81]
							c.1374-2del (SS)	EKCs	[81]
							c.1374-2A>G (SS)	EKCs	[81,88]
							c.1374-1G>A (SS)	EKCs	[81]
							c.1449dup; p.Gly484fs	EKCs	[81]
							c.1546_1551delinsT; p.Gly516fs	EKCs	[91]
							c.1560_1561del; p.Gly521fs	EKCs	[81]
Ichthyosis with confetti type 2, KRT1 (609165)	AD	*KRT1* (139350)	Mitotic recombination	N/A	Loss of heterozygosity of mutant allele	Loss of expression of mutant protein	c.1865dup; p.Val623fs	EKCs	[80]
							c.1759dup; p.Tyr587fs		[87]
							c.591+332_1129-34del; p.197_375del		[89]
Loricrin keratoderma (604117)	AD	*LOR* (152445)	Mitotic recombination	N/A	Loss of heterozygosity of mutant allele	Loss of expression of mutant protein	c.545dup; p.Gly183fs	EKCs	[83]
Keratitis-ichthyosis-deafness syndrome (148210)	AD	*GJB2* (121011)	Second-site mutation	Nucleotide change	Silencing dominant negative allele	Loss of expression of mutant protein	c.148G>A, p.Asp50Asn	EKCs	[82]
Pityriasis rubra pilaris (173200)	AD	*CARD14* (607211)	Mitotic recombination	N/A	Loss of heterozygosity of mutant allele	Loss of expression of mutant protein	c.356T>C; p.Met119Thr	EKCs	[84]
							c.407A>T; p.Gln136Leu	EKCs	[84]

MIM, online Mendelian Inheritance in Man ID (www.omim.org, accessed on 30 June 2022); EB, epidermolysis bullosa; EBS, epidermolysis bullosa simplex; JEB, junctional epidermolysis bullosa; DEB, dystrophic epidermolysis bullosa; RDEB, recessive DEB; DDEB, dominant DEB; AD, autosomal dominant; AR, autosomal recessive; EKCs, epidermal keratinocytes; DFBs, dermal fibroblasts; SS, splice-site; Nt, nucleotide; AA, amino acid.

## 5. Molecular Mechanisms of Revertant Mosaicism in Genodermatoses

**Figure 3:**
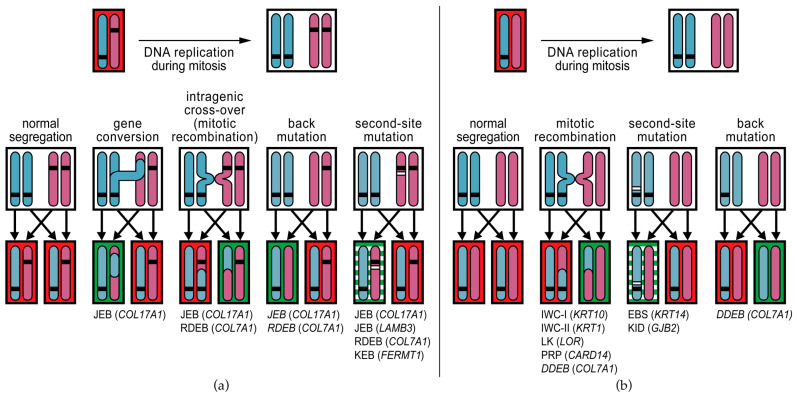
Mechanisms of revertant mosaicism identified in genodermatoses. Different reversion mechanisms can correct pathogenic germline variants. The mechanisms reported in genodermatoses are shown with the diseases indicated below. For every cell (black boxes), one chromosome pair is shown (vertical blue and pink bars); one from the mother, the other from the father. Horizontal black bars on the chromosomes indicate pathogenic germline variants. During mitosis, the DNA of each chromosome is first duplicated to yield two identical sister chromatids. With normal segregation, each daughter cell obtains one sister chromatid from the maternal chromosome and one from the paternal chromosome. (**a**) Mechanisms of reversion reported in autosomal recessive genodermatoses. Gene conversion is the non-reciprocal transfer of DNA from one chromosome to the other. When this occurs in the region where the inherited mutation is located, the mutation is lost in one of the daughter cells, which then only carries one of the recessive mutations and will produce protein (green shading). Intragenic cross-over (a form of mitotic recombination) results in one daughter cell with three inherited mutations, and a revertant daughter cell with only one of the inherited mutations. Second-site mutations indicate additional mutation events (horizontal white bars) on one of the mutant alleles that correct the effect of the pathogenic germline variant. Many different types of second-site mutations have been reported (see Table 1) and the protein that is produced may be similar to the wildtype protein or may be slightly aberrant but still functional (green/white shading). Back-mutation is a form of second-site mutation in which the additional event changes the germline variant back to wildtype. (**b**) In autosomal dominant genodermatoses, where a heterozygous variant causes the disease phenotype, the most prominent reversion mechanism is mitotic recombination. Mitotic recombination is a normal process during DNA replication, but it can lead to cells that carry both mutant alleles and to revertant cells that carry both wildtype alleles. Boxes with red shading indicate mutant cells. Genodermatosis names in italics indicate that it was not possible to distinguish between mitotic recombination and back-mutation as the reversion mechanism. JEB, junctional epidermolysis bullosa; RDEB, recessive dystrophic epidermolysis bullosa; KEB, Kindler epidermolysis bullosa; IWC, ichthyosis with confetti; LK, loricrin keratoderma; PRP, pityriasis rubra pilaris; DDEB, dominant dystrophic epidermolysis bullosa; EBS, epidermolysis bullosa simplex; KID, keratitis-ichthyosis-deafness syndrome.

Over the years, multiple differing mechanisms of somatic correction events have been uncovered (Table 1, Figure 3). The first JEB patient described with RM had a non-affected skin patch in which one of the pathogenic *COL17A1* variants was lost due to a gene conversion [26]. This led to loss of heterozygosity for the *COL17A1* region where one of the pathogenic variants resided and, consequently, the loss of one mutated allele with the restoration of type XVII collagen production and a non-affected skin phenotype.

In subsequent years, various reversion mechanisms have been uncovered for mutations in EB-related genes. Of the different reversion mechanisms described in EB, most involve correction through a somatic, small intragenic mutational event (referred to as second-site mutation) such as somatic point mutations in a germline nonsense codon that revert it to the wildtype or to a different but functional codon [68,75], or alter the splicing of a mutated exon [66,73,74,75,77,79,86]. A special class of additional point mutations is ‘back-mutation’ in which the somatic event reverts the mutant nucleotide to wildtype. Of note, in the cases with presumed back-mutations, it was not always possible to distinguish a back-mutation from a mitotic recombination event [75,86]. Other somatic, small intragenic mutational events that have been reported are deletions or duplications of one or a few nucleotides that either directly restore a disturbed mutant reading frame or modify mutant splicing patterns and thereby restore mutant reading frames [69,70,71,73,78,79]. Such correction events were observed frequently in Kindler EB as certain repetitive nucleotide sequences in *FERMT1* appear to be particularly prone to the duplication or deletion of single nucleotides due to slipped mispairing [69,70]. One revertant nucleotide insertion exerted its corrective effect by disrupting rather than restoring the reading frame [76]. In this case, a nucleotide insertion had occurred on the same allele that carried the dominant germline *KRT14* point mutation, which disrupted the reading frame, thereby silencing the expression of the mutant allele and its dominant-negative effect. A similar phenomenon was observed in the revertant patches of a patient with KID syndrome [82]: five different somatic variants *in cis* with the germline point mutation were found to inhibit the dominant-negative effect of the germline mutations.

Another class of reversion mechanisms involves chromosomal recombination events during mitosis, i.e., gene conversion, intragenic cross-overs, and mitotic recombination. Only a few correction mechanisms identified in EB involve such recombination events [26,64,65,70,72,82,86], probably because most EB patients in which RM has been identified have biallelic gene mutations where recombination events are less likely to create cells without mutations. In contrast, mitotic recombination is the only reversion mechanism so far identified in the autosomal dominant disorders IWC-I, IWC-II, LK, and PRP [80,81,83,84,88,89,90,91]. KID-syndrome is the only autosomal dominant genodermatosis in which a reversion mechanism other than mitotic recombination has been reported (second-site mutation) [82].

The question remains whether there are biological processes that drive the occurrence of RM in genodermatoses. For EB, it is still mostly considered a stochastic process. In contrast, several RM-driving mechanisms are being uncovered for the autosomal dominant genodermatoses. IWC-I, IWC-II, and LK are caused by frameshift mutations in the carboxyl-terminal ends of the *KRT10*, *KRT1*, and *LOR* genes, respectively [80,81,92,93]. These frameshift mutations create arginine-rich tails that subsequently act as nuclear localization signals, causing the mutant proteins to aberrantly mis-localize to the nuclei instead of the cytoplasm and homing to the intermediate filaments or cornified envelope [83,94,95]. The role of this nuclear mis-localization in the pathogenesis of the disturbed keratinocyte differentiation seen in IWC-I, IWC-II, and LK has not been fully elucidated, but it has been suggested that it causes an increased mitotic recombination rate that drives the occurrence of RM [81]. This would explain the high frequency of revertant confetti-like skin patches in patients with these disorders. This hypothesis seems to be supported by the observation of many fewer revertant spots than expected in a patient with a typical IWC-I *KRT10* frameshift mutation, who also carried a heterozygous non-pathogenic pericentric inversion at chromosome 17 (inv(17)(p13q12)) that dramatically reduced the options for therapeutic mitotic recombination events in the *KRT10* chromosomal region (17q21.2) [88]. In PRP, it was shown that mutant CARD14 protein alters the replication stress response in mutant keratinocytes, which preferentially drives break-induced replication as a DNA-repair pathway, resulting in an increased frequency of mitotic recombinations and, consequently, revertant skin spots [84].

## 6. Clinical Recognition of Revertant Mosaicism in Genodermatoses

In genodermatoses, RM appears as non-affected skin patches standing out amidst affected skin (Figure 2). Revertant, non-affected patches are therefore best visible in generalized phenotypes that involve the entire skin, like recessive types of EB or IWC [68,74,75,80,81]. In addition, confirming RM at the tissue level is most reliable in recessive disorders in which the germline mutations cause the complete absence of a structural protein [96]. In such diseases, the re-expression of the structural protein in the revertant patches can be unequivocally demonstrated using immunofluorescence or immunohistochemical staining assays on skin biopsy sections. It can be much more challenging to demonstrate RM in autosomal dominant genodermatoses like dominant DEB (DDEB), in which the expression of the protein involved is normal or only reduced to some extent [86]. In other autosomal dominant conditions, differences in staining patterns between mutant and revertant skin or cultured keratinocytes, rather than differences in expression levels, can help identify RM. For instance, in a case of severe autosomal dominant EB simplex (EBS-severe), RM only became apparent incidentally because a proportion of cultured keratinocytes from an unaffected skin area showed a normal keratin filament network when only mutant cells with filament aggregates were expected [76]. In the autosomal dominant disorders IWC-I, IWC-II, and LK, the mutant keratin 10, keratin 1, and loricrin proteins localize to the nuclei, whereas the revertant proteins integrate normally into the cytoplasmic filament network or cornified envelope [80,81,83].

In addition, patient age plays an important role in the clinical detection of RM. In young children, the skin is usually not yet as much affected, meaning it is difficult to clinically recognize revertant patches if they are present. In IWC, many revertant patches occur and grow with age, which explains why they are easier to recognize with ageing. In EB, however, the gradual skin changes due to cumulative damage with ageing make it easier to recognize revertant patches as patients grow older. Interestingly, we have also studied several clearly unaffected skin patches in EB patients with severe and generalized subtypes later in their lives. We expected these patches to be revertant, but they turned out not to be using either the ‘skin rub test’ or molecular analysis. The skin rub test, which examines the resistance of the skin against mechanical stress by rubbing with the tip of a pen, can discriminate revertant from affected skin (See www.youtube.com/watch?v=fz8nW3z51Gw, accessed 1 June 2022, video courtesy of the late Prof. M.F. Jonkman). However, in patients with milder forms of EB, mutant skin can also be resistant to direct mechanical stress, which makes recognition of revertant patches is even more challenging and sometimes impossible.

The revertant areas do vary in size and shape between the different genodermatoses (Figure 2). For instance, in IWC, the confetti-like white, greenish, or brown revertant areas stand out against the red affected skin [93]. In IWC-I, they appear in childhood and over time increase in number to hundreds and in size from 2–10 mm up to 4 cm [81,88,90,91,93]. In IWC-II, the revertant patches usually do not become apparent until early adulthood and remain smaller in size, 4–15 mm [80,87,89]. The size of the spots is limited by the expansion of the single revertant clone, but they can increase until later in life. This is exemplified by a case reported by Suzuki et al. in which a 56-year-old patient with IWC-II initially presented with non-affected white spots of 1–2 mm that increased up to 15 mm 8 years later [87]. In LK, patients with RM have dozens of well-demarcated, whitish, round, or oval-shaped, non-affected skin patches of up to 10 mm in diameter [83]. In PRP, numerous non-affected skin patches were identified that were up to several centimeters in size [84].

In contrast to IWC, LK, and PRP, the revertant areas in EB are usually larger and irregular in shape. In addition, changes in size and shape during adulthood have not been witnessed in patients with intermediate JEB due to *COL17A1* mutations [79]. The exact size of the revertant area that originates from a single clone is not clear, as different revertant populations of keratinocytes, each originating from a different reversion event, have sometimes been found in a single revertant area. In intermediate JEB, the patches are especially recognizable after sun exposure because of differences in pigmentation, as type XVII collagen is also involved in melanocyte supply to the epidermis [97]. In the affected areas there is a decreased melanocyte density compared to the revertant areas. In addition, the skin texture of the revertant skin patches was shown to be smooth when compared to the coarse affected skin in a *COL17A1*-associated JEB-intermediate patient [63]. Only two patients with intermediate JEB due to *LAMB3* mutations and RM have been described in the literature, and both had multiple revertant areas with at least one patch carrying two separate reversion events [74]. One of the patients claimed that the revertant area on his lower leg healed to clinically unaffected skin later in life, but there was no photo material available to confirm this. The revertant areas in RDEB are usually more difficult to recognize than those in patients with intermediate JEB, especially compared to patients with mutations in *COL17A1*. The revertant areas in DEB can vary in size from 3 × 3 cm to 10 × 5 cm, and their pigmentation is usually normal [65,68,71,72,86]. Lastly, in Kindler EB, the revertant areas are numerous and smaller compared to the other major EB types, varying from 0.5 cm^2^ to 3 cm^2^ [48,69,70]. Larger areas up to 15 cm^2^ in size were noted in some patients, but it could not be excluded that these originated from overlapping areas arising from separate reversion events. In one of the patients, the revertant area did not change in size over a 3-year period. One possible explanation for the high number of revertant areas is that these cells have a significant growth advantage compared to their affected counterparts, as mutant kindlin-1 severely impacts keratinocyte proliferation [70].

## 7. Revertant Mosaicism as Treatment for Genodermatoses

Currently, no truly curative gene-therapy treatments for genodermatoses have been approved, and treatment is usually symptomatic. Nonetheless, there has been much progress with disease-modifying treatments, some of which have led to dramatic improvements in the severity of certain genodermatoses [98,99,100,101,102,103,104,105]. The holy grail for curing genodermatoses is likely a genome-editing method capable of effectively and safely correcting the causative gene variants in the entire body. However, genome-editing poses several challenges including off-target editing effects, the need to target enormous numbers of stem cells on many different body locations, and the difficulty of reaching the genomic DNA in all these cells, which requires vehicle methods that might carry their own risks [106]. In addition, there are ethical issues around genome-editing [107]. Hence, although the first clinical studies have commenced [108], these issues may prevent genome-editing from entering the clinic as a routine systemic treatment for genodermatoses in the short run.

Most approaches under study for EB therefore focus on topical treatment [109], either by topical compound delivery (e.g., *in vivo* gene-addition by viral [110] or minicircle non-viral vectors [111], antisense oligonucleotides [112]), *ex vivo* gene-replacement or gene-correction followed by injection of corrected fibroblasts [113], or the transplantation of large epidermal sheets cultured from *ex vivo*-corrected keratinocytes [114,115,116,117], analogous to the treatment of full-thickness burn-wounds [118]. In 2017, Hirsch et al. reported a 7-year-old boy with laminin 332-deficient JEB in whom almost the entire epidermis (~0.85 m^2^) was replaced using exogenously corrected, autologous skin grafts [119,120]. Keratinocyte stem cells obtained from a 4 cm^2^ skin biopsy were first transduced with a retroviral vector expressing the full-length *LAMB3* cDNA, then cultured into large transgenic epidermal grafts that were transplanted onto properly prepared dermal wound beds.

It is in this context that it is especially interesting to look more closely at the phenomenon of RM. In RM, nature has successfully achieved what science so eagerly strives for: the complete cure of a genodermatosis in a specific skin area by safely repairing the effect of the germline mutation(s). Therefore, ever since the first discovery of RM in EB in 1997, our group has been looking for ways to acquire revertant skin cells from revertant patches and to expand them and use them to treat affected skin areas. Using naturally corrected, patient-own skin cells would have three major advantages: (i) no need for potentially dangerous gene-correction technologies, (ii) the potential for permanent correction, and (iii) no risk of immuno-rejection of revertant skin transplants or grafts. Our group showed a proof-of-concept for the therapeutic potential of revertant keratinocytes in 2014. Seventy-three 3 mm punch biopsies from a revertant, laminin 332-positive skin patch were transplanted as mini-grafts onto seven persistent ulcers of a patient with laminin 332-deficient JEB [121]. In 18-month follow-up, no blisters were observed and laminin 332-expression was increased in the treated areas, while the revertant donor site also healed with revertant keratinocytes, indicating that the revertant skin cells were able to settle and expand *in vivo*. Despite these promising results, taking so many biopsies from the revertant skin areas can still only treat relatively small affected areas. Following the full-body transplantation method introduced by Hirsch et al. [120], an abstract to the 2021 Society of Investigative Dermatology Meeting reported the replacement of ~80% of the epidermis of an RDEB patient by epidermal autografts generated from cultured revertant keratinocytes obtained from a revertant skin patch [122]. This provides evidence that revertant keratinocyte stem cells have sufficient proliferative potential to regenerate large areas of epidermis *in vitro*. Although undoubtedly highly beneficial to the respective patients, the question remains how sustainable this invasive procedure would be in routine practice, as it requires repeated and multiple operations usually under general anesthesia [120]. In addition, the durability of the transplanted revertant skin is another unknown factor.

**Figure 4:**
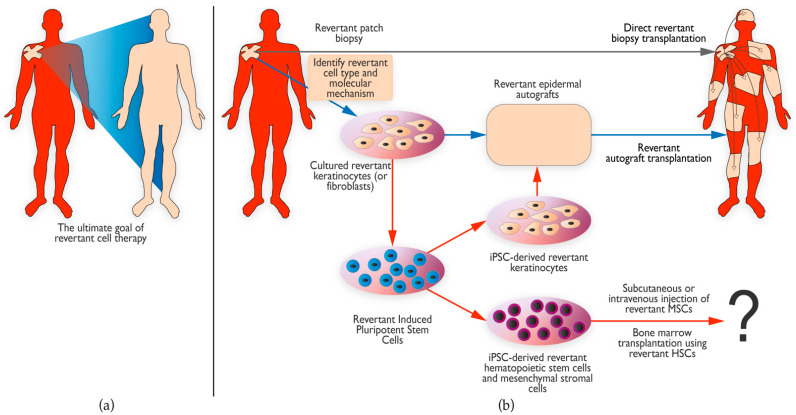
Therapeutic prospects of revertant mosaicism. (**a**) The ultimate goal of revertant cell therapy is to use the patient’s own naturally corrected cells to cure the entire skin. (**b**) Several attempts have already been made to use revertant skin cells for treatment, such as direct transplantation of small revertant biopsies to an affected skin area (grey arrow) [121]. Others have focused on first expanding the revertant cells to produce larger revertant epidermal grafts that were then transplanted onto larger areas (pathway indicated by blue arrows) [122]. Lastly, induced pluripotent stem cells (iPSCs) can be generated from revertant cells to provide an unlimited supply of revertant cells (iPSC pathways indicated by red arrows). These iPSCs can be subsequently differentiated into keratinocytes to form revertant epidermal autografts [126]. Another possibility is to differentiate these iPSCs into hematopoietic stem cells (HSCs) or mesenchymal stromal cells (MSCs) that could be used for bone marrow transplantation [67] or subcutaneous or intravenous injection, respectively. The question mark indicates that these possibilities have not yet been pursued on patients and the outcome of such approaches is thus unknown.

Induced pluripotent stem cells (iPSCs) have been proposed as a therapeutic route for genodermatoses and have been generated from patients with EBS [123], JEB [124], and RDEB [123,125], among others. When revertant cells are used to create iPSCs, genome-editing to correct the mutations is not required, which is a major advantage. Two groups have reported successful generation of iPSCs from revertant keratinocytes. Umegaki-Arao et al. generated iPSCs from revertant keratinocytes from a patient with JEB due to *COL17A1* mutations [126]. The revertant iPSCs were trans-differentiated into keratinocytes that expressed type XVII collagen. The authors showed that they were able to generate three-dimensional epidermal equivalents *in vitro* and could reconstitute human epidermis from these iPSC-derived keratinocytes *in vivo* in mice, and that type XVII collagen localized to the basement membrane zone. Tolar et al. generated iPSCs from revertant keratinocytes of a patient with RDEB due to *COL7A1* mutations [67] that they were able to trans-differentiate into epidermal and hematopoietic cell populations. These studies showed that revertant keratinocytes can be a viable source for developing iPSC-based therapeutic approaches in genodermatoses. iPSC-derived revertant keratinocytes could serve as an unlimited source of cells for the generation of autografts for transplantation, while iPSC-derived revertant hematopoietic cells could be envisioned in combination with bone marrow transplantation strategies [127]. Whether iPSC-derived revertant keratinocytes possess the growth potential to be cultured into large grafts to treat larger skin areas remains to be seen. While the use of revertant cells in such approaches may circumvent some of the limitations of genome-editing methods, the safety of iPSC-based treatments has been debated. Obstacles that were mentioned for clinical use were carcinogenicity, a lack of *in situ* integration, genomic instability, and/or immunologic rejection [128]. Progress is, however, being made in the stem cell research community to produce safe methods for the generation and expansion of human pluripotent stem cells. Kim et al. showed that in 2022, there were 19 ongoing therapeutic clinical trials with iPSC strategies, most of them being conducted in Japan (10 trials), but none were yet focused on skin diseases [129]. In addition, the iPSCs by Umegaki-Arao et al. [126] and Tolar et al. [67] were generated using retroviral vectors and therefore cannot be used in a clinical situation, as this strategy carries a risk of uncontrolled genomic integration [130]. Figure 4 summarizes the therapeutic approaches using revertant skin cells that have already been performed and the ones that may be pursued in the future.

## 8. Concluding Remarks and Future Perspectives

RM is a form of natural gene therapy that occurs more often than previously thought in the skin of patients with genodermatoses. Multiple genetic mechanisms have been found to underlie the reversions to ‘normal’ phenotypes, the nature of which depends on the mode of inheritance and the type of germline mutation. As revertant patches may provide a source for future revertant cell therapy, it is becoming increasingly important to identify these revertant patches on a patient’s body. Clinicians should therefore be on the lookout for patches of skin that are not affected by the phenotype of the respective genodermatosis. The great advantage of using revertant skin cells in therapeutic approaches is that these autologous cells do not require additional genetic modifications. Several *in vitro* and even *in vivo* pilot studies have already indicated that revertant cells may provide a source for therapy for the severe genetic skin blistering disease EB. However, several crucial questions need to be addressed before revertant skin cells can be used as an efficient and safe therapy in patients. How can we identify revertant patches *in vivo* in young patients, where affected skin is much more difficult to differentiate from revertant skin? How can we efficiently identify and isolate revertant stem cells from a revertant skin patch? What are the characteristics of these revertant stem cells? Additionally, do they differ from mutant stem cells from the same patients and healthy controls? Finally, how can we introduce these revertant stem cells back into the patients in the most efficient and least burdensome way? These will be topics for future studies that will hopefully lead to RM as a therapeutic avenue for patients with rare, currently incurable genodermatoses.

## Data Availability

Not applicable.

## Supplementary Materials

The following supporting information can be downloaded at https://www.mdpi.com/article/10.3390/biomedicines10092118/s1, Supplementary Table S1: Genetic conditions in which revertant mosaicism has been reported in tissues other than skin.
