# Supplementary material for: Revertant Mosaicism in Genodermatoses: Natural Gene Therapy Right before Your Eyes
Source: Biomedicines. 2022 Aug 29;10(9):2118. doi: 10.3390/biomedicines10092118 (PMC9495737; doi:10.3390/biomedicines10092118)
Supplement: Supplementary file 1 [file biomedicines-10-02118-s001.zip › biomedicines-1820934-supplementary.pdf]

Table S1. Genetic conditions in which revertant mosaicism has been reported in other tissues than skin.

Disease group	Disease	OMIM	Corrected gene	OMIM	References
Metabolic	Lesch-Nyhan syndrome	300322	*HPRT1*	308000	[61]
Metabolic	Tyrosinemia type I	276700	*FAH*	613871	[58]
Metabolic	Familial amyloidotic polyneuropathy	105210	*TTR*	176330	[36]
Neuromuscular	Duchenne muscular dystrophy	310200	*DMD*	300377	[60]
Neuromuscular	Myotonic dystrophy	160900	*DMPK*	605377	[59]
Neuromuscular	Hereditary motor and sensory neuropathy type 1A	118220	*PMP22 duplication*	601097	[55]
Neurologic, hematologic	Ataxia pancytopenia	159550	*SAMD9L*	611170	[37]
Immunologic	Adenosine deaminase deficiency	102700	*ADA*	608958	[56]
Immunologic	X-linked severe combined immunodeficiency	300400	*IL2RG*	308380	[54]
Immunologic	Omenn syndrome	603554	*RAG1*	179615	[49]
Immunologic	T-cell immunodeficiency	610163	*CD3-zeta (CD247)*	186780	[48]
Immunologic	Leukocyte adhesion deficiency type 1	116920	*ITGB2*	600065	[46]
Immunologic	Autosomal recessive severe combined immunodeficiency	608971	*IL7R*	146661	[42]
Immunologic	Autosomal recessive severe combined immunodeficiency	600802	*JAK3*	600173	[43]
Immunologic	Hyper-IgE recurrent infection syndrome, autosomal recessive	243700	*DOCK8*	611432	[41]
Immunologic	Omenn syndrome	603554	*CARD11*	607210	[40]
Immunologic	WHIM syndrome	193670	*CXCR4*	162643	[38]
Immunologic	Immunodeficiency 25	610163	*CD247*	186780	[35]
Immunologic	X-linked lymphoproliferative syndrome type 1	308240	*SH2D1A*	300490	[28]
Immunologic, dermatologic	X-linked hypohidrotic ectodermal dysplasia with immunodeficiency	300291	*IKBKG*	300248	[50]
Hematologic, immunologic	Wiskott-Aldrich syndrome	301000	*WAS*	300392	[52]
Hematologic, immunologic	Bone marrow failure syndrome 4	618116	*MYSM1*	612176	[39]
Hematologic, immunologic	Platelet-abnormalities with eosinophilia and immune-mediated inflammatory disease	617718	*ARPC1B*	604223	[31]
Hematologic	Fanconi anemia, complementation group C	227645	*FANCC*	613899	[53]
Hematologic	Fanconi anemia, complementation group A	227650	*FANCA*	607139	[51]
Hematologic	Fanconi anemia, complementation group N	610832	*PALB2*	610355	[45]
Hematologic	Fanconi anemia, complementation group I	609053	*FANCI*	611360	[47]
Hematologic	Diamond-Blackfan anemia 10	603701	*RPS26*	613309	[33]
Hematologic	Monosomy 7 myelodysplasia and leukemia syndrome	252270	*SAMD9L*	611170	[34]

Hematologic	Fanconi anemia, complementation group B	300514	*FANCB*	300515	[32]
Hematologic	Diamond-Blackfan anemia 1	105650	*RPS19*	603474	[29]
Hematologic, dermatologic	Dyskeratosis congenita, autosomal dominant 1	127550	*TERC*	602322	[44]
Multi-system	Bloom syndrome	210900	*RECQL3*	604610	[57]
Multi-system	MIRAGE syndrome	617053	*SAMD9*	610456	[30]
Multi-system	Shwachman-Diamond syndrome	260400	*SBDS*	607444	[27]
